# Calpain‐1 mediates vascular remodelling and fibrosis via HIF‐1α in hypoxia‐induced pulmonary hypertension

**DOI:** 10.1111/jcmm.17295

**Published:** 2022-04-01

**Authors:** Haiyan Deng, Xiaoxue Tian, Hening Sun, Huan Liu, Meili Lu, Hongxin Wang

**Affiliations:** ^1^ 154516 Key Laboratory of Cardiovascular and Cerebrovascular Drug Research of Liaoning Province Jinzhou Medical University Jinzhou China

**Keywords:** calpain‐1, fibrosis, HIF‐1α, hypoxic pulmonary hypertension, vascular remodelling

## Abstract

Calpain‐1, a calcium‐activated neutral cysteine proteases, has been reported to be involved in the formation of pulmonary hypertension. HIF‐1α, an oxygen‐sensitive transcription factor, has been reported to activate genes involved in cell proliferation and extracellular matrix recombination. This study was designed to investigate the effect of calpain‐1 in hypoxic pulmonary hypertension (HPH) and to explore whether there is a relationship between calpain‐1 and HIF‐1α in this disease. In the hypoxia‐induced model of HPH, we found that hypoxia resulted in increased right ventricular systolic pressure, right ventricular hypertrophy, pulmonary vascular remodelling and collagen deposition in lung tissues of mice. The levels of calpain‐1 and HIF‐1α were up‐regulated in the lung tissues of hypoxia‐treated mice and pulmonary arterial smooth muscle cells (PASMCs). Knock‐out of calpain‐1 restrained haemodynamic and histological changes induced by chronic hypoxia in mice, and inhibition of calpain‐1 also repressed the abnormal proliferation and migration of PASMCs. Besides, knock‐out or inhibition of calpain‐1 suppressed hypoxia‐induced expression of HIF‐1α, VEGF, PCNA, TGF‐β1, MMP2 and collagen I in vivo and in vitro. While inhibition of HIF‐1α abolished the above effects of calpain‐1. Furthermore, we found that calpain‐1 mediates the expression of HIF‐1α through NF‐κB (P65) under hypoxia conditions. In conclusion, our results suggest that calpain‐1 plays a pivotal role in hypoxia‐induced pulmonary vascular remodelling and fibrosis through HIF‐1α, providing a better understanding of the pathogenesis of HPH.

## 1 INTRODUCTION

Pulmonary hypertension (PH) is a fatal vascular disease defined as pulmonary vascular resistance (PVR) > 3 wood units associated with mean pulmonary arterial pressure (mPAP) ≥20 mm Hg at rest.^1^ According to the most recent clinical classification of PH, Hypoxic pulmonary hypertension (HPH) belongs to the third category of this disease and characterized by pulmonary arteries remodelling, which include arterial fibrosis, hyperproliferation and abnormal apoptosis.^1^, ^2^ HPH is a vascular disease caused by continuous exposure to hypoxic environment. Chronic hypoxia can lead to abnormal contraction and remodelling of pulmonary vessels, increase pulmonary artery pressure and finally lead to right heart failure and death.^3^, ^4^ Up to now, the exact pathogenesis of HPH is still unclear, and there is a lack of effective treatment in clinic. Therefore, it is necessary to find new potential therapeutic targets for HPH.

The calpain system has been discovered for more than 50 years and is expressed in multiple organs of human body.^5^ Calpains belong to the calcium‐dependent non‐lysosomal neutrocysteine endopeptidyase family and include 15 isoforms. Among them, calpain‐1 and calpain‐2 are two major typical calpain proteinases, which are composed of 80 kD large subunits with catalytic activity and 28 kD small subunits with regulatory activity.^6^, ^7^ Calpains have been found to be involved in the pathological process of pulmonary hypertension (PH). For example, Wan et al^8^. showed that the imbalance of extracellular calpain/calpastatin is involved in the pathological process of PH. In addition, Zhang et al^9^. found that calpain‐1 expression is significantly up‐regulated in rat pulmonary arteries under hypoxic conditions, which promotes abnormal proliferation of PASMCs and participates in the development of HPH. However, the mechanism of how calpain induces the proliferation of PASMCs has not been clarified. In addition, calpain has been found to mediate the synthesis of Collagen I by promoting the synthesis and activation of TGF‐β1 in bleomycin‐induced pulmonary fibrosis.^10^

Hypoxia‐inducible factor‐1 (HIF‐1) is composed of the following two subunits: a stably expressed β subunit and an O_2_‐regulated α subunit. Under low oxygen conditions, HIF‐1α accumulates and transferred to the nucleus, combines with HIF‐1β to form a heterodimer, transcribes and activates genes related to energy metabolism, cell proliferation, apoptosis and extracellular matrix recombination.^11^, ^12^ Mo et al^13^. found that HIF‐1α overexpression increases VEGF expression and calpain activity in human umbilical vein endothelial cells, leading to angiogenesis. Besides, studies have shown that HIF‐1α is critical for TGF‐β1‐induced Collagen I synthesis in glomerulosclerosis.^14^ Previous studies in our lab have shown that calpain‐1 mediates IκBα degradation and NF‐κB activation in heart tissue.^15^ In addition, studies have shown that P65 is one of the key transcription factors in PASMCs that promote HIF‐1α protein accumulation.^16^

According to the above research status, we hypothesized that calpain‐1 is involved in hypoxia‐induced pulmonary vascular remodelling and fibrosis in a HIF‐1α dependent manner, hoping to provide a new theoretical basis for calpain‐1 as a potential therapeutic target of HPH.

## 2 MATERIALS AND METHODS

### 2.1 Animal experiments

Animal experiments were conducted in accordance with the guidelines of Animal Care and Use Committee of Jinzhou Medical University (2021018). C57BL/6 mice were acquired from Liaoning changsheng biotechnology co. LTD. Calpain‐1(−/−) C57BL/6 mice were obtained from Cyagen (Guangzhou) Biotechnology Co. LTD. The mice were randomly assigned to the following six groups (*n* = 8 per group): WT Normoxia, KO Normoxia, WT Hypoxia, KO Hypoxia, Hypoxia+YC‐1 and Hypoxia+BAY11‐7082. The mice in hypoxia+YC‐1 and hypoxia+BAY11‐7082 groups were separately intraperitoneal injection with YC‐1 (1 mg/kg/day, Selleck) and BAY11‐7082 (5 mg/kg, 3 times/week, Selleck). The mice in normoxia groups were exposed to normal environment containing 21% O_2_, while those in hypoxia groups were exposed to a normobaric chamber containing 10% O_2_ for 4 weeks.^17^ After 4 weeks, the mice were anaesthetized with 1% pentobarbital sodium (40 mg/kg i.p.). Carefully detach the right external jugular vein and the return blood flow was blocked with a fine line. Then, the Miller catheter connected to the biofunctional experimental system was slowly inserted through an incision cut in the right external jugular vein to measure right ventricular systolic pressure (RVSP).^18^ The collected serum and cardiopulmonary tissues were stored in a −80°C refrigerator for further experiments.

### 2.2 Culture and treatment of primary rat PASMCs

SD rats were anesthetized with pentobarbital sodium and soaked with 75% alcohol for disinfection. The pulmonary arteries were then isolated in a sterile environment. After scraping the inner and outer membranes, the middle membranes were cut into small pieces and transferred to cell culture vials. DMEM (Gibco) supplemented with 20% foetal bovine serum (FBS, Sigma) and 1% penicillin/streptomycin was added and cultured in an incubator (37°C, 5% CO_2_). After 3–5 days, cells can be observed around the tissue mass. After 8–10 days, the cells were digested and subcultured with 0.25% trypsin‐EDTA (Genview). The cells were identified as PASMCs by immunofluorescence staining with α‐SMA. Subsequent in vitro experiments divided PASMCs into the following five groups: Normoxia, Hypoxia, Hypoxia+MDL28170 (10 μmol/L), Hypoxia+YC‐1 (10 μmol/L) and Hypoxia+BAY11‐7082 (10 μmol/L). Cells from the normoxic group were cultured in a normal incubator (37°C, 5% CO_2_, 95% ambient air), while cells from the hypoxic group were cultured in a hypoxic incubator (37°C, 3% O_2_, 5% CO_2_).

### 2.3 Histology analysis

The lungs of mice in each group were fixed with 4% paraformaldehyde for 48 h, embedded in paraffin after gradient ethanol, and cut into 4‐μm‐thick sections for further experiment. The paraffin sections were stained by haematoxylin‐eosin (HE) staining and Masson staining, respectively, to observe vascular remodelling and fibrosis. The percentage of pulmonary artery wall thickness to total thickness (WT/TT)% and the percentage of wall area to total area (WA/TA)% were calculated and analysed by Image‐Pro Plus to evaluate pulmonary artery remodelling.^3^

### 2.4 Determination of right ventricular hypertrophy

The hearts excised from the mice were washed with PBS and divided into two parts: right ventricle (RV) and left ventricle plus interventricular septum (LV+S), and their dry weights were weighed, respectively. The degree of right cardiac hypertrophy was calculated and evaluated according to right ventricular hypertrophy index: (RV/[LV+S]).^19^

### 2.5 Immunohistochemical staining

After routine dewaxing and hydration, the sections were subjected to high‐pressure antigen repair, and then incubated with 3% H_2_O_2_, blocked with goat serum and incubated overnight with primary antibody Ki‐67 (1:100, Beyotime) at 4°C. The next day, the second antibody was incubated in darkness, and then DAB (ZSGB‐BIO) was used to visualize, observed and interpreted under light microscope.

### 2.6 Immunofluorescence staining

Pulmonary arterial smooth muscle cells cultured in 24‐well plates were fixed with 4% paraformaldehyde dissolved in PBS and then permeabilized with 0.3% triton X‐100. After blocking with 5% BSA, primary antibodies against α‐SMA (1:100, ABclonal), calpain‐1 (1:100, proteintech) and P65 (1:200, proteintech) were incubated overnight at 4°C. On the second day, FITC labelled Goat Anti‐Rabbit IgG or FITC labelled Goat Anti‐Mouse IgG (1:200, ABclonal) was incubated in darkness and the nucleus were stained with DAPI (Beyotime). The images were observed and captured with a fluorescence microscope.

### 2.7 Western blot assay

Lung tissues and PASMCs were lysed in radio immunoprecipitation assay (RIPA) lysis buffer (containing 1% PMSF) on ice to extract total proteins. Nuclear and cytoplasmic proteins were extracted following the manufacturer's recommendations of the nuclear and cytoplasmic extraction kit (Beyotime). After protein quantification, the same amount of protein from each sample were separated on sodium dodecyl sulphate‐polyacrylamide gel electrophoresis (SDS‐PAGE), and then the proteins on the gel were transferred to PVDF membranes (Millipore) by semi‐dry electrophoretic transfer system (Bio‐rad). The membranes were then blocked with 5% BSA and incubated with primary antibodies against calpain‐1 (1:1000, proteintech), HIF‐1α (1:1500, ABclonal), P65 (1:2000, proteintech), β‐actin (1:5000, proteintech), Lamin B (1:10000, proteintech), VEGF (1:1000, ABclonal), TGF‐β1 (1:800, Wanleibio), PCNA (1:1500, ABclonal), MMP2 (1:1000, ABclonal) and collagen I (1:1000, Wanleibio) overnight at 4°C. On the second day, membranes were incubated with HRP‐conjugated secondary antibodies (1:10000, ABclonal). The immune response bands were visualized with a chemiluminescence reagents (Biosharp).

### 2.8 ELISA assay

TGF‐β1 levels in the serum of mice were determined with a mouse TGF‐β1 ELISA kit (mlbio) following the manufacturer's recommendations.

### 2.9 Cell proliferation and migration assay

Pulmonary arterial smooth muscle cells were inoculated into 96‐well plates and cultured in hypoxic or normoxic incubators for 24 h. Cell proliferation was tested by CCK‐8 kit (Abmole) following the manufacturer's instructions. Transwell migration was conducted in a 24‐well transwell chamber (Corning). The cells were fixed in 4% paraformaldehyde dissolved in PBS and then stained with 0.1% crystal violet. Those unmigrated cells in upper chamber were removed, and the images of migrated cells in lower chamber were taken with a light microscope, five areas were randomly selected for cell counting.

### 2.10 Statistical analysis

All data were presented as means ± SD, and GraphPad Prism (version 8.0.2) was used for statistical analysis. One‐way analysis of variance (ANOVA) was used to test the statistical differences among the experimental groups. *p* < 0.05 was statistically significant.

## 3 RESULTS

### 3.1 Expression of calpain‐1 in mice model of HPH

**FIGURE 1:**
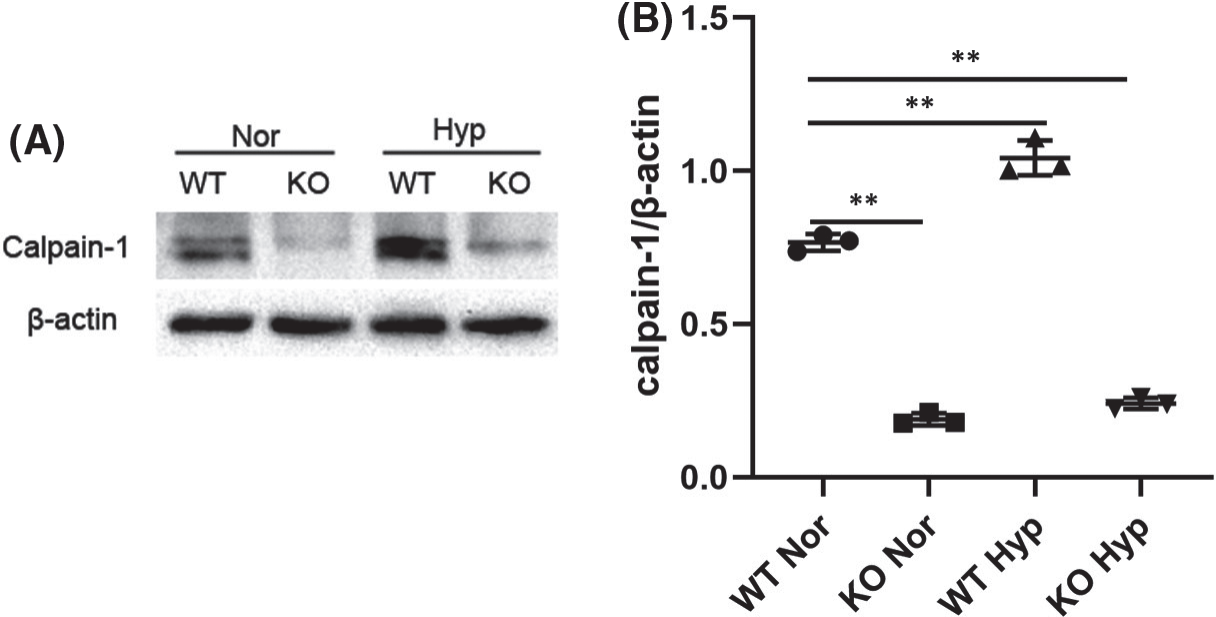
Expression of calpain‐1 in mice model of HPH. (A) The protein levels of calpain‐1 in lung tissues was detected by Western blot assay. (B) Relative grey values of the protein bands were shown. Data were presented as mean ± SD. **p < 0.01 versus the WT Normoxia group

To demonstrate whether calpain‐1 is involved in the pathogenesis of HPH, Western blot analysis of lung tissues was performed to assess calpain‐1 protein levels in C57BL/6 mice treated with hypoxia or normoxia for 4 weeks. As illustrated in Figure 1A,B, compared with normoxic group, calpain‐1 levels in the lung tissues of hypoxic mice were significantly increased. To reduce the level of calpain‐1, calpain‐1 was knocked out in mice. Compared with WT mice, the calpain‐1 level of KO mice was significantly decreased in lung tissues.

### 3.2 Calpain‐1 is involved in hypoxia‐induced pulmonary vascular remodelling and fibrosis

**FIGURE 2:**
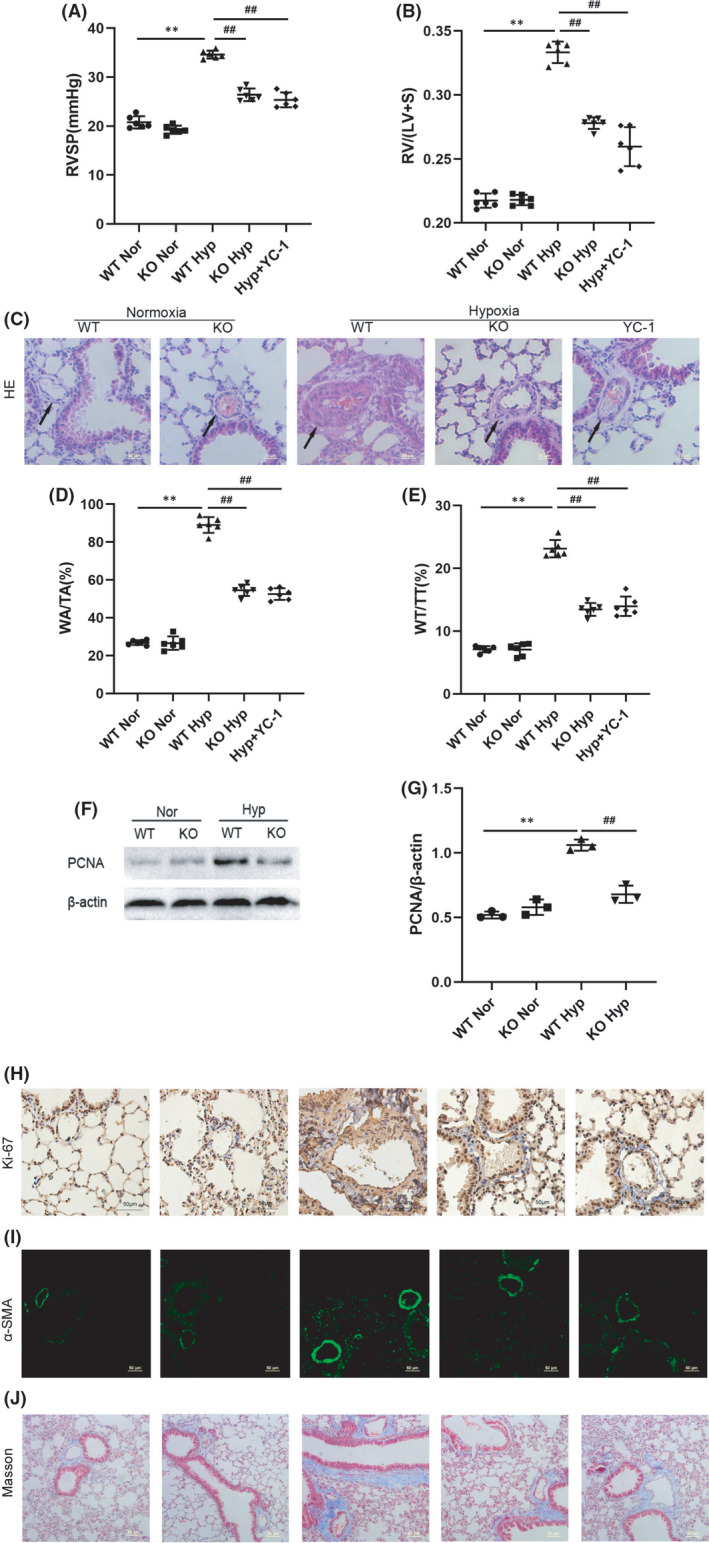
Calpain‐1 is involved in hypoxia‐induced pulmonary vascular remodelling and fibrosis. The RVSP (A) and RV/(LV+S; B) of mice in different groups were shown. (C) HE staining was used to observe the vascular morphology of lung tissues. (D, E; WA/TA) and (WT/TT) values for each group were analysed. (F) The protein levels of PCNA in lung tissues was detected by Western blot assay. (G) Relative grey values of the protein bands were shown. (H) The expression of Ki‐67 in lung tissues was assessed by immunohistochemical staining. (I) The expression of α‐SMA in lung tissues was assessed by immunofluorescence staining. (J) Pulmonary fibrosis was detected by Masson's staining. Data were presented as mean ± SD. **p < 0.01 versus the WT Normoxia group, ^##^ *p* < 0.01 versus the WT Hypoxia group

Haemodynamic and vascular remodelling parameters were measured in C57BL/6 mice treated with hypoxia or normoxia for 4 weeks. As shown in Figure 2A‐E, hypoxia significantly increased RVSP, right ventricular hypertrophy and vessel wall thickness in WT mice following 4 weeks of exposure, whereas no significant pathological changes were observed in KO mice. Besides, Western blot was used to analyse PCNA expression in lung tissues of mice. In addition, analyses of Ki‐67, α‐SMA and the formation of collagen fibre were conducted using paraffin‐embedded mice lung tissues. The expression of PCNA, Ki‐67, α‐SMA and the formation of collagen fibre in the lung tissues of hypoxia group were significantly increased compared with the control group, whereas knock‐out of calpain‐1 could significantly alleviate the above changes of HPH mice (Figure 2F‐J). These results indicated that calpain‐1 was involved in vascular remodelling and fibrosis in mice with hypoxia‐induced pulmonary hypertension.

### 3.3 Effects of calpain‐1 on proliferation and migration of hypoxia‐treated PASMCs

**FIGURE 3:**
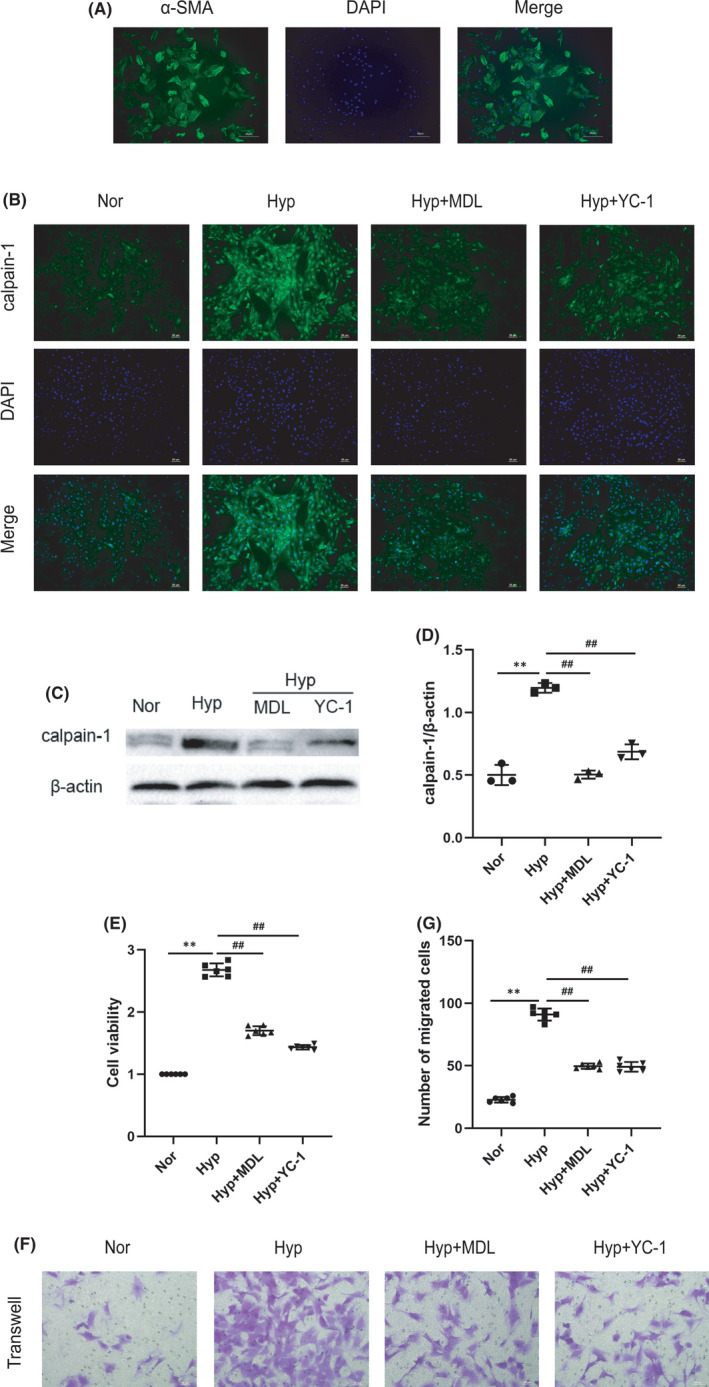
Effect of calpain‐1 on hypoxia‐induced proliferation and migration of PASMCs. (A) Immunofluorescence staining of α‐SMA. (B) The expression of calpain‐1 in PASMCs was assessed by immunofluorescence staining. (C) The protein levels of calpain‐1 in PASMCs was detected by Western blot assay. (D) Relative grey values of the protein bands were shown. (E) CCK‐8 was used to detect the cell viability. (F) PASMCs migration was detected by Transwell assay. (G) The migrated cells was counted and analysed. Data were presented as mean ± SD. **p < 0.01 versus the normoxia group, ^##^ *p* < 0.01 versus the hypoxia group

Primary PASMCs were extracted from rat pulmonary arteries and identified by α‐SMA (Figure 3A), then exposure to hypoxia. As illustrated in Figure 3B‐D, hypoxia treatment significantly increased calpain‐1 levels in PASMCs, while calpain‐1 levels were significantly inhibited by calpain‐1 inhibitor MDL28170. As shown in Figure 3E‐G, the proliferation and migration of PASMCs were detected by CCK‐8 and transwell assays respectively. The results showed that the proliferation and migration of PASMCs were significantly enhanced after hypoxia, while MDL28170 inhibited them.

### 3.4 Calpain‐1 promoted the expression of proliferation and fibrosis‐related molecules through HIF‐1α

**FIGURE 4:**
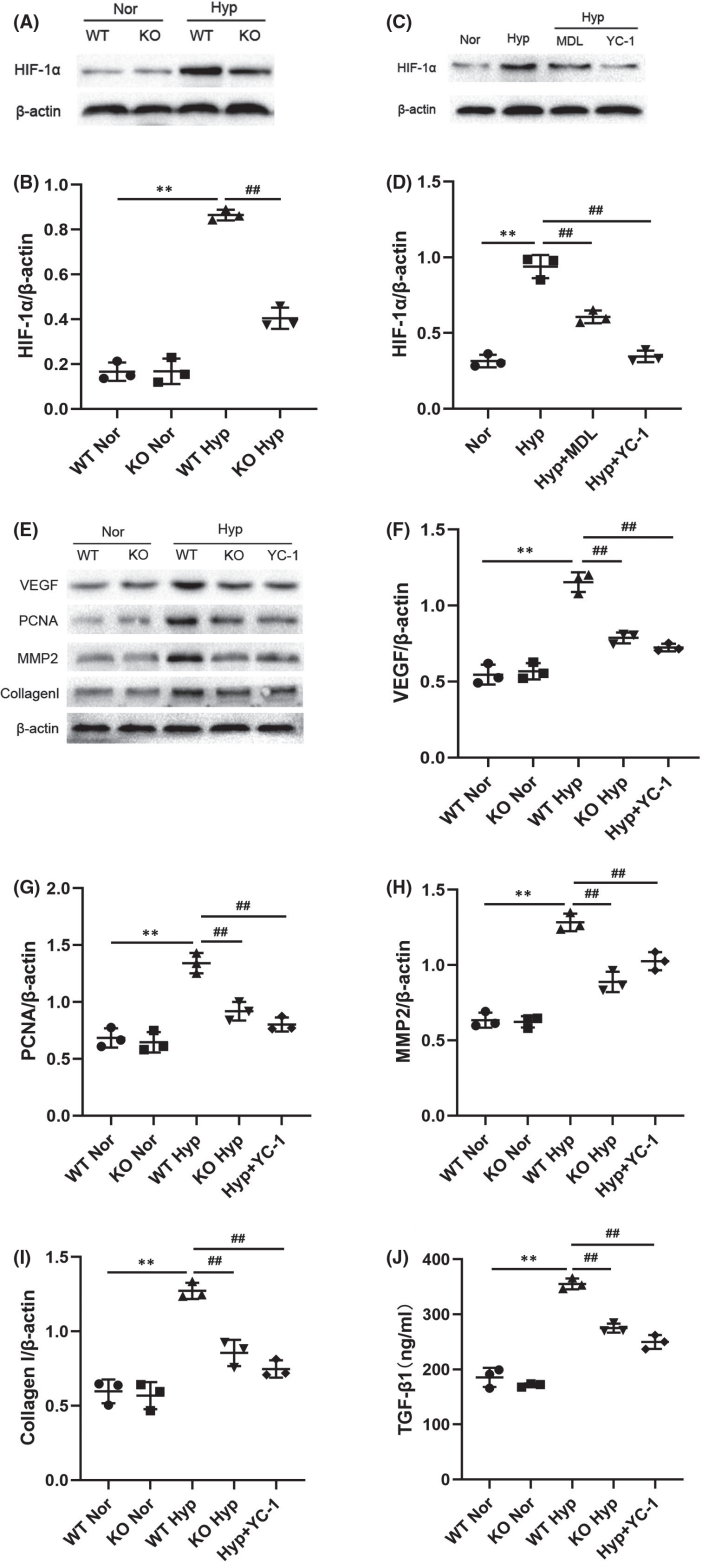
Calpain‐1 promoted the expression of proliferation and fibrosis‐related molecules through HIF‐1α. (A) The protein levels of HIF‐1α in lung tissues was detected by Western blot assay. (B) Relative grey values of the protein bands were shown. (C) The protein levels of HIF‐1α in PASMCs was detected by Western blot assay. (D) Relative grey values of the protein bands were shown. (E) The protein levels of VEGF, PCNA, MMP2 and collagen I in lung tissues were detected by Western blot assay. (F)‐(I) Relative grey values of the protein bands were shown. (J) The levels of TGF‐β1 in serum of mice was quantified by ELISA. Data were presented as mean ± SD. **p < 0.01 versus the normoxia group, ^##^ *p* < 0.01 versus the hypoxia group

**FIGURE 5:**
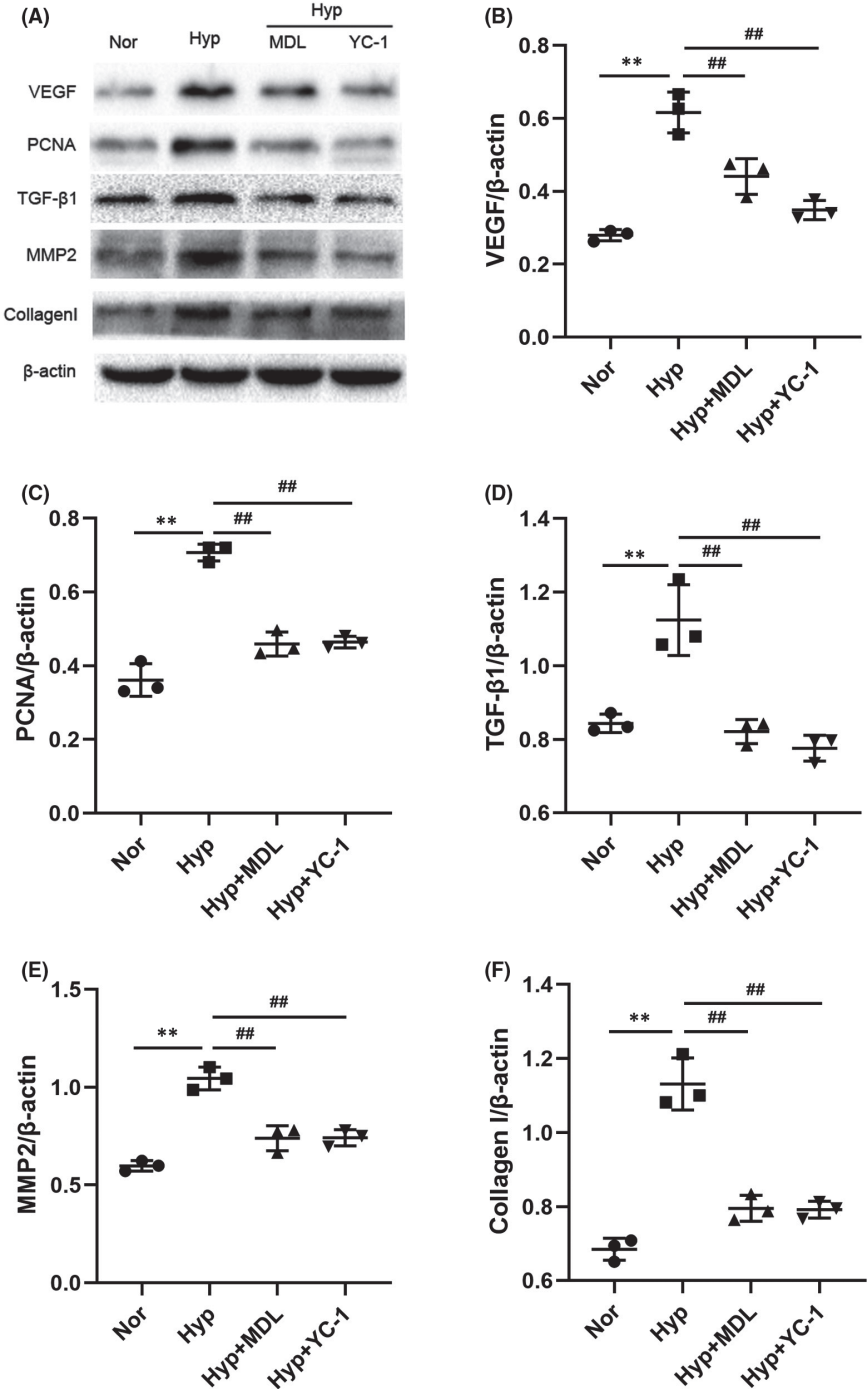
Calpain‐1 promoted the expression of proliferation and fibrosis‐related molecules through HIF‐1α. (A) The protein levels of VEGF, PCNA, TGF‐β1, MMP2 and collagen I in PASMCs were detected by Western blot assay. (B‐F) Relative grey values of the protein bands were shown. Data were presented as mean ± SD. **p < 0.01 versus the normoxia group, ^##^ *p* < 0.01 versus the hypoxia group

We have just demonstrated that the protein level of calpain‐1 increases significantly under hypoxic conditions. To investigate the role of calpain‐1 in HIF‐1α‐mediated functions, Western blot was used to detect the expression levels of HIF‐1α, PCNA, VEGF, TGF‐β1, MMP2 and Collagen Ⅰ in lung tissues and PASMCs. The results of Western blots (Figure 4A‐D) showed that hypoxia increased the level of HIF‐1α, while the level of HIF‐1α decreased after calpain‐1 knock‐out or inhibition. Consistent with the changes in HIF‐1α, the increase of calpain‐1 dramatically promoted the expression of VEGF, PCNA, TGF‐β1, MMP2 and Collagen Ⅰ in lung tissues, while inhibition of HIF‐1α by YC‐1 abolished these effects of calpain‐1 (Figure 4E‐J). The results in vitro were consistent with in vivo experiments (Figure 5A‐F). In addition, haemodynamic and vascular remodelling parameters were also consistent with the above changes (Figure 2A‐J). Moreover, inhibition of HIF‐1α by YC‐1 abolished the effect of calpain‐1 on promoting cell proliferation and migration in PASMCs (Figure 3E‐G). The above experimental results indicating that HIF‐1α may be a downstream mediator of the effects of calpain‐1, and calpain‐1 regulates proliferation and fibrosis‐related proteins in a HIF‐1α‐dependent manner.

### 3.5 Calpain‐1 mediates the expression of HIF‐1α through NF‐κB under hypoxia conditions

**FIGURE 6:**
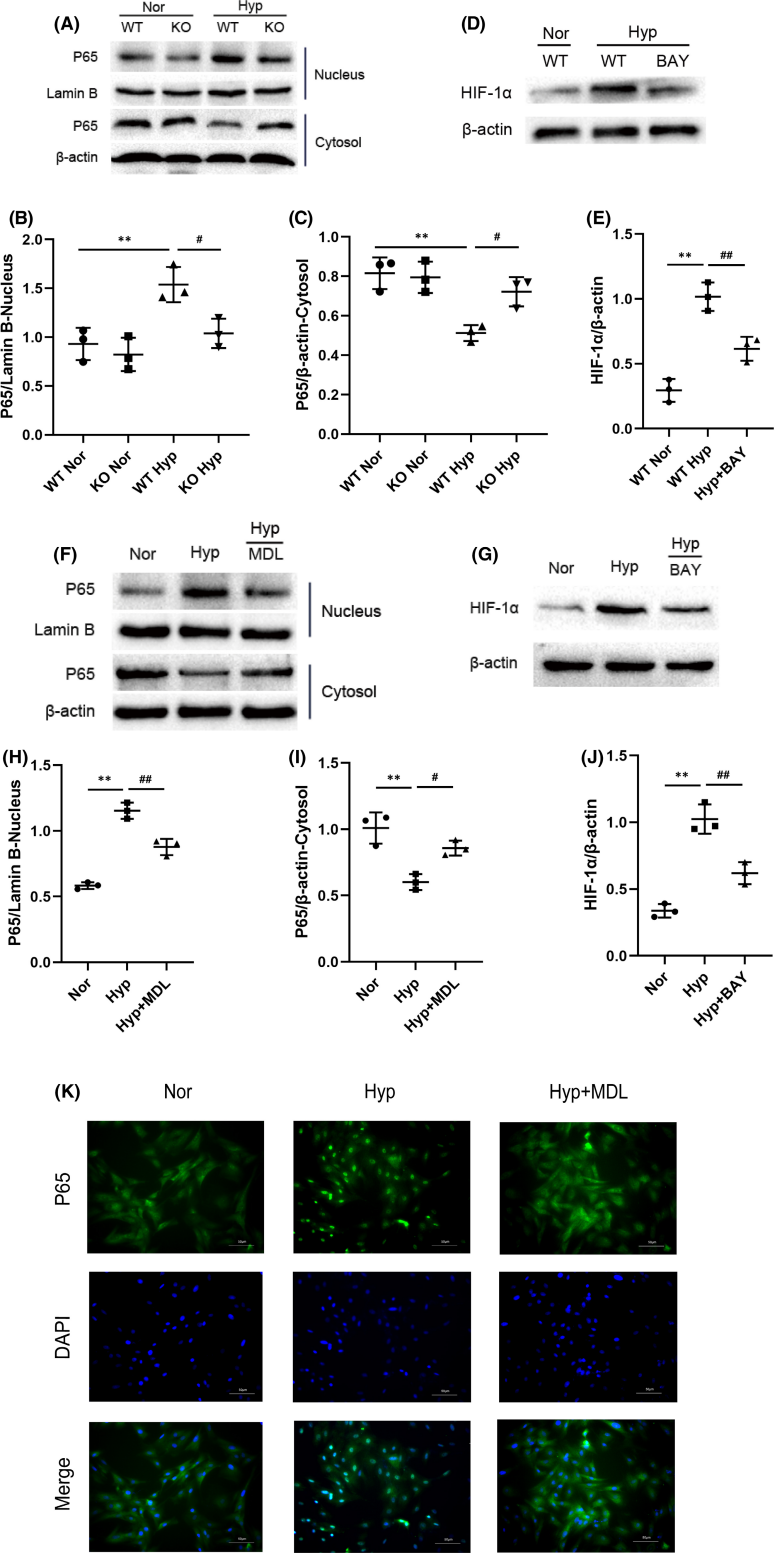
Calpain‐1 mediates the expression of HIF‐1α through NF‐κB under hypoxia conditions. (A) The protein levels of P65 in the nucleus and cytosol of lung tissues were detected by Western blot assay. (B, C) Relative grey values of the protein bands were shown. (D) The protein levels of HIF‐1α in the lung tissues were detected by Western blot assay. (E) Relative grey values of the protein bands were shown. (F) The protein levels of P65 in the nucleus and cytosol of PASMCs were detected by Western blot assay. (G, H) Relative grey values of the protein bands were shown. (I) The protein levels of HIF‐1α in PASMCs were detected by Western blot assay. (J) Relative grey values of the protein bands were shown. (K) The expression of P65 in PASMCs was assessed by immunofluorescence staining. Data were presented as mean ± SD. **p < 0.01 versus the normoxia group, ^##^ *p* < 0.01 versus the hypoxia group

Based on the observation that calpain‐1 regulates the protein expression of HIF‐1α, we further explored the mechanism of how calpain‐1 triggers HIF‐1α expression in lung tissues and PASMCs. Previous studies in our laboratory have shown that calpain‐1 promotes the activation of NF‐κB in heart tissue. In addition, studies have shown that NF‐κB is one of the key transcription factors in PASMCs that promote HIF‐1α protein accumulation. We next investigated whether NF‐κB (P65) is involved in calpain‐1 mediated HIF‐1α expression in HPH mice lung tissues and PASMCs under hypoxia. As shown in Figure 6A‐C, hypoxia can lead to the increased expression of nuclear protein P65 in the lung tissues of WT mice, but no significant changes were observed in KO mice. Besides, inhibition of NF‐κB expression by BAY11‐7082 attenuated the promotion effect of calpain‐1 on HIF‐1α expression (Figure 6D,E). Meanwhile, the same results were observed in PASMCs (Figure 6F‐K).

### 3.6 A positive feedback loop exists between calpain‐1‐HIF‐1α axes during hypoxia

**FIGURE 7:**
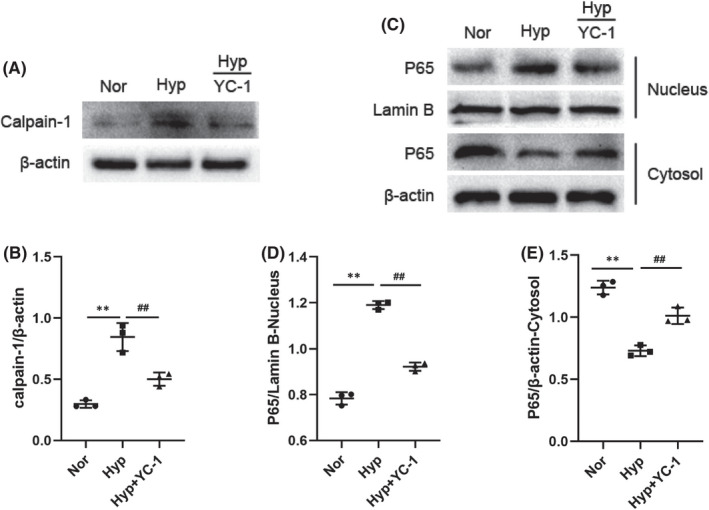
Positive feedback loop exists between calpain‐1 and HIF‐1α signalling during hypoxia. (A) The protein levels of calpain‐1 was detected by Western blot assay. (B) Relative grey values of the protein bands were shown. (C) The protein levels of P65 in the nucleus and cytosol of PASMCs were detected by Western blot assay. (D, E) Relative grey values of the protein bands were shown. Data were presented as mean ± SD. **p < 0.01 versus the normoxia group, ^##^ *p* < 0.01 versus the hypoxia group

To further explore the relationship between calpain‐1 and HIF‐1α, the expression of HIF‐1α in PASMCs was inhibited by YC‐1. IF and Western blot results indicated that inhibition of HIF‐1α decreased hypoxia‐induced calpain‐1 expression (Figure 3B and Figure 7A,B). Besides, pharmacological inhibition of HIF‐1α signalling pathway also attenuated the effect of calpain‐1 on nuclear protein P65 expression during hypoxia (Figure 7C‐E), indicating that HIF‐1α is responsible for hypoxia and calpain‐1‐induced nuclear protein P65 secretion. These results indicated that a positive feedback loop exists between HIF‐1α and calpain‐1/NF‐κB signalling during hypoxia.

## 4 DISCUSSION

In this study, the mice were exposed to a normobaric chamber containing 10% O_2_ for 4 weeks to develop a model of pulmonary hypertension as previously reported.^17^ We found for the first time that cross‐regulation between calpain‐1 and HIF‐1α is a key process that promotes the development of HPH. Furthermore, we found that calpain‐1 interacts with HIF‐1α in vivo and in vitro by activating NF‐κB (P65) under hypoxia, promoting the development of HPH by regulating the expression of proteins related to cell proliferation and fibrosis. Moreover, inhibition of HIF‐1α significantly reduced the expression of calpain‐1 and nuclear protein P65.

Growing studies have shown that calpain is involved in many diseases, including cardiovascular diseases, cancer, neurodegenerative diseases and several other diseases.^20^, ^21^, ^22^, ^23^, ^24^ Consistent with previous studies,^9^ our results showed that hypoxia elevated calpain‐1 protein levels in both lung tissues of mice with HPH and PASMCs, significantly increased RVSP, right ventricular hypertrophy and vessel wall thickness, increased the expression of PCNA, Ki‐67, α‐SMA and the formation of collagen fibre in the lung tissues, and enhanced the proliferation and migration of PASMCs. While knock‐out or inhibiting the expression of calpain‐1 reversed the above changes.

It has been reported that calpain‐1 is involved in the formation of fibrosis by regulating the expression of angiopoietin‐1, IL‐6, MMP2, collagen I and III. Besides, calpain‐1 overexpression can also induce activation of TGF‐β1/Smad signalling pathway in vascular smooth muscle cells.^25^, ^26^ We then examined the levels of proteins associated with proliferation and fibrosis in the HPH mice and PASMCs under hypoxia. Consistent with previous studies,^9^, ^18^, ^27^, ^28^ we found that hypoxia up‐regulated VEGF, PCNA, TGF‐β1, MMP2 and Collagen I levels in vivo and in vitro, while knock‐out or inhibiting the expression of calpain‐1 down‐regulated the levels of these proteins.

Studies have demonstrated that chronic hypoxia results in the stable expression of HIF‐1α, and HIF‐1α upregulates the expression of hypoxia response genes. These genes mediate changes in cell proliferation, migration and extracellular matrix (ECM) deposition, which are the main mechanisms promoting vascular remodeling.^29^, ^30^ Wu et al^31^. found that enriched environment inhibited the degradation of STAT3 by inhibiting the activity of calpain‐1, thus activating the subsequent STAT3/HIF‐1α/VEGF signalling pathway and promoting post‐stroke neurorepair and functional recovery. Besides, Zhou et al^32^. found that in addition to the established proteasome degradation pathway, the disruption of HIF‐1α by calpain is another pathway affecting HIF‐1α protein content in renal cell carcinoma (RCC4) cells. However, Zheng et al^33^. found that calpain activity was up‐regulated under hypoxia, resulting in cleavage of filamin A (FLNA), generating a fragment that promoted nuclear localization of HIF‐1α, which was corecruited to HIF‐1α target gene promoters, ultimately leading to HIF‐1α target gene expression increased in hypoxia‐dependent manner in tumour. On the basis of these studies, we investigated the relationship between calpain‐1 and HIF‐1α in rat PASMCs and mice with hypoxic pulmonary hypertension. Our study showed that hypoxia increased HIF‐1α levels, while HIF‐1α levels decreased after calpain‐1 knock‐out or inhibition, which was consistent with Zheng et al.^33^ In addition, when we inhibited HIF‐1α with YC‐1 abolished the promoting effects of calpain‐1 on haemodynamic, vascular remodelling parameters, VEGF, PCNA, TGF‐β1, MMP2, Collagen Ⅰ and proliferation and migration of PASMCs. All the results indicated that HIF‐1α may be a downstream mediator of the effects of calpain‐1.

Our further results suggested that NF‐κB (P65) could be involved in calpain‐1 mediated HIF‐1α expression in vivo and in vitro. Tian et al^34^. found that IκBα is an important substrate of calpain, and proteolysis of IκBα leads to activation of NF‐κB in cardiac tissues. Studies have shown that activation of P65 leads to activation of HIF‐1α promoter activity, which increases HIF‐1α mRNA level and promotes HIF‐1α protein accumulation.^16^, ^35^ Besides, overexpression of NF‐κB also resulted in increased HIF‐1α protein expression even under normoxic environments in human embryonic kidney (HEK)‐293 cell.^36^ In this study, we found that hypoxia resulted in an calpain‐1‐dependent increase in nuclear protein P65 expression. In addition, inhibition of NF‐κB expression with BAY11‐7082 significantly attenuated the promotion effect of calpain‐1 on HIF‐1α expression. Further research found that inhibition of HIF‐1α reduced the expression of calpain‐1 induced by hypoxia, which also attenuated the effect of calpain‐1 on nuclear protein P65 expression during hypoxia, indicating that a positive feedback loop exists between HIF‐1α and calpain‐1/NF‐κB signalling during hypoxia.

In conclusion, our results provide experimental evidence that the calpain‐1‐ HIF‐1α axis plays a pivotal role in hypoxia‐induced vascular remodelling and fibrosis, and provide a new theoretical basis for calpain‐1 as a potential therapeutic target for HPH.

## CONFLICT OF INTEREST

All authors claimed that there was no conflict of interest in the study.

## AUTHOR CONTRIBUTIONS

**Haiyan Deng:** Data curation (equal); Methodology (equal); Software (equal); Validation (equal); Writing – original draft (equal); Writing – review & editing (equal). **Xiaoxue Tian:** Methodology (equal). **Hening Sun:** Investigation (equal). **Huan Liu:** Software (equal). **Meili Lu:** Data curation (equal); Funding acquisition (equal). **Hongxin Wang:** Funding acquisition (equal); Project administration (equal).

## Data Availability

The data used to support the findings of this study are available from the corresponding author upon request.
